# hTAC internalizes via both clathrin-dependent and clathrin-independent endocytosis in mammalian cells

**DOI:** 10.1007/s13238-018-0508-9

**Published:** 2018-03-17

**Authors:** Xinyu Zhu, Min Li, Xiaojun Xu, Rui Zhang, Xiaofei Zhang, Zhuo Ma, Jingze Lu, Tao Xu, Junjie Hou, Eli Song

**Affiliations:** 1 Key Laboratory of Molecular Biophysics of the Ministry of Education, College of Life Science and Technology, Huazhong University of Science and Technology, Wuhan, 430074 China; 2 National Laboratory of Biomacromolecules, CAS Center for Excellence in Biomacromolecules, Institute of Biophysics, Chinese Academy of Sciences, Beijing, 100101 China; 3 College of Life Sciences, University of Chinese Academy of Sciences, Beijing, 100049 China

**Dear Editor,**

Endocytosis is a crucial process employed by cells to internalize nutrients and turnover membrane components and is essential for many functions, including nutrient uptake, signal transduction, cytokinesis, morphogenesis, cell adhesion and migration. Endocytosis is classified as clathrin-dependent endocytosis (CDE) or clathrin-independent endocytosis (CIE) according to its dependence on clathrin. Several different CIE pathways have been proposed, including caveolin-dependent endocytosis, flotillin-dependent endocytosis, the clathrin-independent carrier pathway, ARF6-dependent endocytosis, phagocytosis, macropinocytosis, the IL2Rβ pathway (Doherty and McMahon, 2009), the newly identified fast endophilin-mediated endocytosis pathway (Boucrot et al., 2015) and the EGFR-NCE pathway (Caldieri et al., 2017).

CDE has been extensively studied in recent decades. Thus, the molecular mechanism and physiological function of CDE have been gradually revealed. The CIE pathways are typically defined by undisturbed internalization in the absence of clathrin or the endocytic morphological criteria. However, the mechanism and functional significance of CIE remain largely unknown. This lack of understanding is potentially due to the complexity of the different CIE endocytosis pathways and the lack of cargo makers in the CIE pathways. The α chain of the interleukin-2 receptor (IL-2Rα, also termed hTAC) is considered a CIE cargo because its internalization was initially determined to be independent of dynamin and showed no co-localization with clathrin on the plasma membrane (PM) (Naslavsky et al., 2003). hTAC has also been shown to be recycled to the PM by tubular endosomes in an ARF6-dependent manner (Radhakrishna and Donaldson, 1997; Naslavsky et al., 2003). Furthermore, hTAC has been employed in numerous studies to investigate the CIE pathway in *C*. *elegans* intestine cells and mammalian cells. These studies identified many CIE regulators, including ARF6, EHD1/RME1, TAT-1, Hook1, SEC10, RAB10, EHBP-1, DNPP-1, TBC-2, AMPH-1 and Erbin (Chen et al., 2014; Mayor et al., 2014; Liu et al., 2017). Although hTAC has been widely used as a CIE cargo to investigate the CIE pathway and related endosomal trafficking, knowledge regarding the molecular mechanism underlying hTAC endocytosis and trafficking remains limited partially because of the lack of systematic technology. Furthermore, the poor understanding of the molecular mechanism of hTAC hinders its use as a model to explain the CIE pathway and related mechanism of intracellular trafficking.

In this study, to extensively explore the molecules involved in the endocytosis and intracellular trafficking of hTAC, we employed the APEX2 proximity labeling approach (Rhee et al., 2013) to map the potential proteins involved in hTAC internalization and trafficking in living cells. APEX2 was genetically targeted to the C-terminus of hTAC to orient APEX2 towards the cytosol (Fig. S1A). The hTAC-APEX2 fusion protein was expressed in U2OS cells; hTAC-APEX2 correctly localized on the PM or in the cytosol, and the fusion of APEX2 to hTAC did not disturb hTAC endocytosis (Fig. S1B and S1C). Subsequently, living cells were treated with H_2_O_2_ for 1 min in the presence of BP. APEX2 catalyzes the oxidation of BP to generate a very short-lived biotin-phenoxyl radical that covalently tags the endogenous proteins near hTAC-APEX2 (<20 nm) (Fig. S1A). The efficiency of the biotinylation by hTAC-APEX2 was determined by performing both an immunofluorescence assay and Western blotting. As shown in Fig. S1C–D, APEX2 produced a robust labeling pattern in living U2OS cells treated with substrates (H_2_O_2_ and BP). Then, the biotinylated proteins were isolated with streptavidin-coated beads and separated by SDS-PAGE (Fig. S1E). The proteins were then digested in gels and identified by MS (Table S1).

Interestingly, several CDE regulators, including AP2A1, AP2B1, CLTC, HIP1R, DBNL, MYO6, PIK3C2A and PICALM (Fig. 1A), were identified during the hTAC-APEX2 mapping. Because hTAC is considered a CIE cargo, we were interested in determining whether hTAC could indeed interact with CDE-related proteins. We selected AP2 and CLTC, which are the key effectors in the CDE pathway, and performed co-immunoprecipitation assays to detect their interactions with hTAC. As shown in Fig. 1B, CLTC, AP2A1 and AP2B1 could be immunoprecipitated by hTAC, but the immunoprecipitated bands were weak, likely because the interactions are transient and labile (Salazar et al., 2009). Therefore, CLTC/AP2A1/AP2B1 may be involved in hTAC endocytosis.

**Figure 1:**
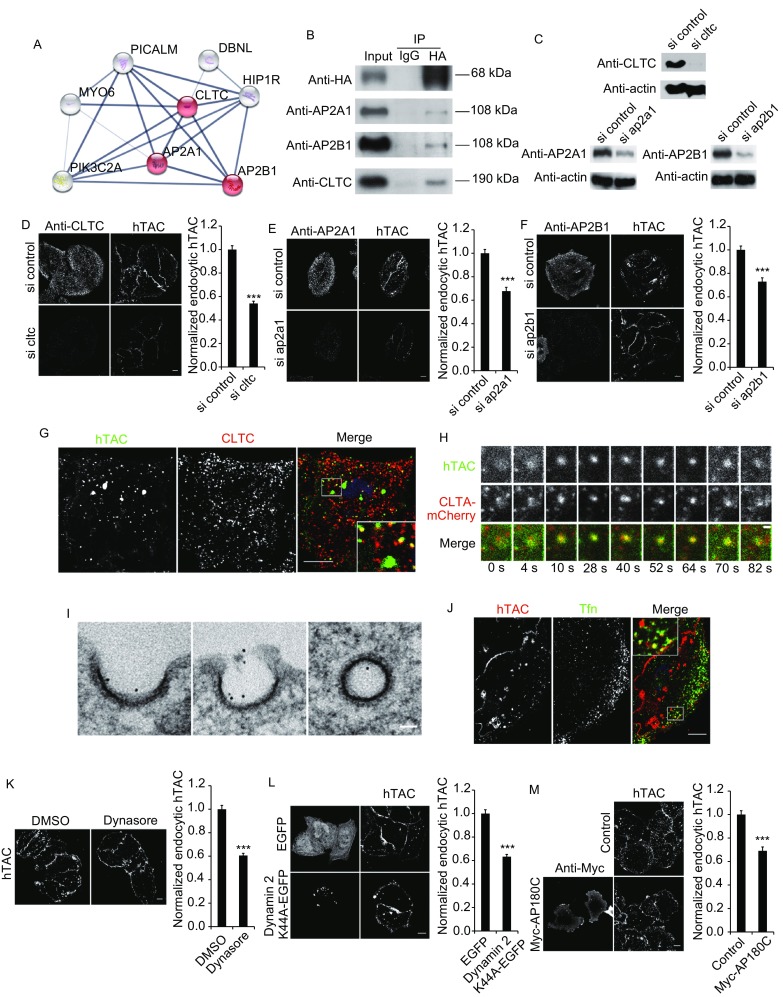
**hTAC can be internalized by the CDE pathway**. (A) Interaction network of known CDE-related proteins identified by hTAC-APEX2 mapping. The highlighted proteins (AP2A1, AP2B1 and CLTC) were selected for further confirmation in subsequent experiments. This network Figure was acquired from the STRING website (http://www.string-db.org/). (B) Immunoblotting of the elution from the co-immunoprecipitation with an anti-HA antibody in hTAC-HeLa cell lysates. The results showed that CLTC, AP2A1 and AP2B1 could interact with hTAC. (C) Immunoblot representing the efficiency of the AP2A1, AP2B1 and CLTC knockdown with siRNA. (D–F) Knockdown of AP2A1, AP2B1 or CLTC decreases hTAC endocytosis. In each sub-Figure, the left column shows a representative image of the respective assay, and the right column shows the quantification of the assay. Three independent replicates of the AP2A1, AP2B1 and CLTC knockdown were performed in each experiment. The following numbers of cells were collected for the statistical analysis: for CLTC shown in (D), *n* = 79/82 (control/si cltc) cells; for AP2A1 shown in (E), *n* = 81/81 (control/si ap2a1) cells; and for AP2B1 shown in (F), *n* = 99/137 (control/si ap2b1) cells. *** Indicates *P* < 0.001, Student’s *t*-test. Scale bars shown in (D–F), 10 µm. (G) Endocytic hTAC co-localized with CLTC in small puncta. The white square is enlarged for a magnified view. Scale bar, 10 µm. (H) Representative TIRFM image of living hTAC-HeLa cells transfected with CLTA-mCherry and treated with an EGFP-FKBP working solution. The results showed that hTAC and clathrin appeared and disappeared synchronously at the PM. Scale bar, 1 µm. (I) EM images showed that Qdot-labeled hTAC localized in CCPs and CCVs. Scale bar, 50 nm. (J) The co-localization of endocytic hTAC and Tfn was observed in hTAC-HeLa cells. The white square is enlarged for a magnified view. Scale bar, 10 µm. (K) The dynamin inhibitor dynasore decreased hTAC endocytosis. The left column shows a representative image of the experiment, and the right column shows the quantification of the assay. Three independent replicates were performed. *n* = 67/102 (DMSO/Dynasore) cells. *** Indicates *P* < 0.001, Student’s *t*-test. Scale bar, 10 µm. (L) Transfection with dynamin 2 K44A-EGFP decreased hTAC endocytosis. The left column shows a representative image of the experiment, and the right column shows the quantification of the assay; three independent replicates were performed. *n* = 94/96 (EGFP/Dynamin 2 K44A-EGFP) cells. *** Indicates *P* < 0.001, Student’s *t*-test. Scale bar, 10 µm. (M) Transfection with the CDE pathway inhibitor Myc-AP180C decreased hTAC endocytosis. The left column shows a representative image of the experiment, and the right column shows the quantification of the assay; three independent replicates were performed. *n* = 99/94 (control/Myc-AP180C) cells. *** Indicates *P* < 0.001, Student’s *t-* test. Scale bar, 10 µm

Next, we used a small interfering RNA (siRNA)-induced knockdown to further analyze the participation of CLTC/AP2A1/AP2B1 in hTAC endocytosis. To probe hTAC endocytosis, we employed the newly developed labeling PM proteins with recombinant FKBP-tagged fluorescent proteins (LAPREP) approach (Zhang et al., 2014) (Fig. S2A), which can be used to detect hTAC endocytosis and is superior to approaches using an antibody (Fig. S2B and S2C). We generated a stable mono HeLa cell clone expressing low FRBT2098L-HA-hTAC (hereafter called hTAC-HeLa), which was used as a cell model in the subsequent experiments. The knockdown of CLTC, AP2A1 and AP2B1 led to a significant inhibition of hTAC endocytosis (Fig. 1C–F). Therefore, these results imply that hTAC can be internalized via the CDE pathway in addition to the previously reported CIE pathway.

To confirm the involvement of the CDE pathway in hTAC endocytosis, we examined the involvement of clathrin in hTAC endocytosis. Endocytic hTAC co-localized with clathrin in small puncta, but not in patch structures, in fixed cells (Fig. 1G), and hTAC and clathrin appeared and disappeared synchronously on the PM in living cells as determined under a total internal reflection fluorescence microscope (TIRFM) (Fig. 1H). hTAC was also detected in the assembly and development of clathrin-coated vesicles by electron microscopy (EM) (Fig. 1I). Moreover, endocytic hTAC was co-localized with the well-known CDE cargo Tfn in small puncta (Fig. 1J). Thus, that the CDE pathway participates in hTAC endocytosis.

Subsequently, we used the chemical inhibitor dynasore and the dominant-negative mutant of dynamin 2 to detect hTAC endocytosis when the CDE pathway was inhibited by the loss of the function of dynamin 2, which is a key factor in the scission of clathrin-coated pits from the PM. Following the suppression of the function of dynamin 2 by either dynasore or dynamin 2 K44A, hTAC endocytosis was reduced (Fig. 1K and 1L). In addition, hTAC endocytosis was inhibited by the overexpression of AP180C, which is an effective inhibitor of the CDE pathway (Fig. 1M). These results show for the first time that hTAC can be internalized by the CDE pathway, even though hTAC was previously considered a CIE cargo.

EHD1 has been previously reported to induce recycling tubules containing the CIE cargo MHC I in an ARF6-dependent manner in HeLa cells (Caplan et al., 2002), and the worm ortholog RME-1 was found to regulate hTAC recycling at the basolateral pole by endosomal tubules in *C*. *elegans* intestine cells (Chen et al., 2014). Here, we observed that EHD1 co-localized with hTAC in both tubules and punctate structures in HeLa cells (Fig. 2A), although not all cells contained the hTAC tubules. Under normal conditions, the proportion of cells containing hTAC tubules was approximately 25% (Fig. 2B), which is consistent with a recent report (Dutta and Donaldson, 2015). Following the treatment of hTAC-HeLa cells with cytochalasin B (CB), which is an F-actin polymerization inhibitor that blocks the recycling of hTAC and preserves recycling tubules, the proportion of cells containing tubules increased to approximately 85% (Fig. 2B). This finding is consistent with previous observations (Radhakrishna and Donaldson, 1997).

**Figure 2:**
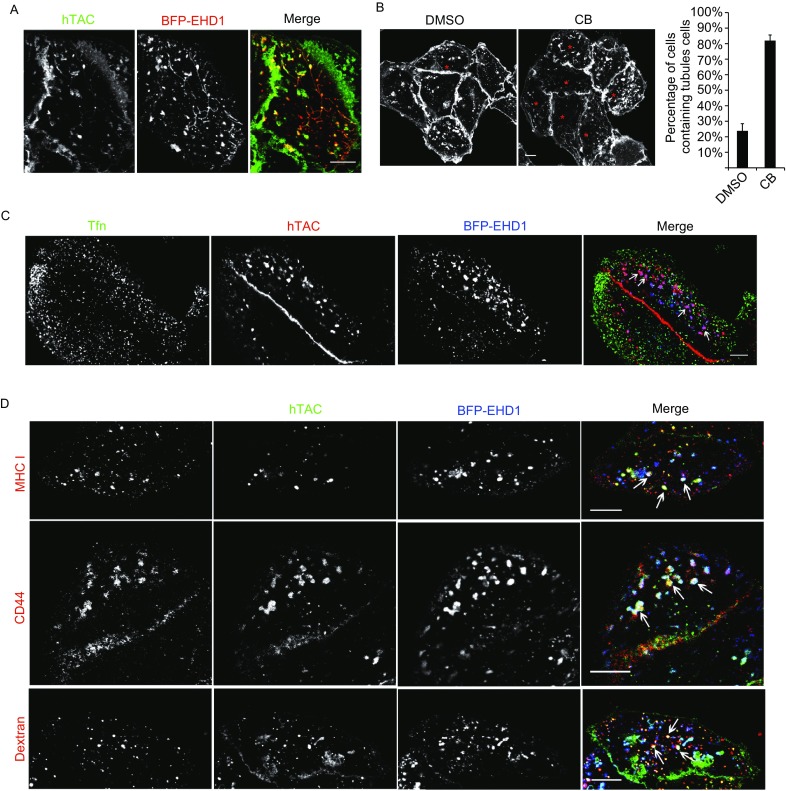
**hTAC was ingested into both tubular structures and patch-like endosomal structures**. (A) Endocytic hTAC co-localized with BFP-EHD1 in tubules and punctate structures. Scale bar, 10 µm. (B) CB treatment increased the percentage of cells containing tubules (red asterisk). The left column shows a representative image, and the right column summarized the percentage of cells containing hTAC tubules. Seven independent replicates were performed. *n* = 571/606 (DMSO/CB) cells. Scale bar, 10 µm. (C) hTAC, but not Tfn, localized in the BFP-EHD1-positive patch-like structures (arrowheads). Scale bar, 10 µm. (D) hTAC co-localized with CD44, MHC I and dextran in the BFP-EHD1-positive patch-like structures (arrowheads). Scale bar, 10 µm

Many hTAC proteins were observed in large punctate structures (designated patch-like structures) at the subplasmalemmal plane in most cells (Fig. 2A and 2B). These patch-like structures were exquisitely labeled by EHD1 (Fig. 2A). The patch-like structures did not contain the CDE cargo Tfn (Fig. 2C) but contained MHC I (Fig. 2D), a well-known CIE cargo. The patch-like structures also contained other well-known CIE cargoes, including CD44 and dextran (Fig. 2D). Therefore, hTAC can be internalized by the CIE pathway.

hTAC was internalized into the EHD1-positive patch-like endosomal structures rapidly; endocytic hTAC could be detected in these structures in less than 15 s (Fig. S3A and Movie 1). Moreover, the patch-like structures were very dynamic, as rich tubulo-vesicular structures continuously budded from and fused to other structures (Fig. S3B and Movie 2), which is similar to previously reported observations in *C*. *elegans* (Chen et al., 2014).

ARF6 has been previously shown to co-localize with endocytic hTAC in the tubular endosome and regulate hTAC recycling. Here, we demonstrated that ARF6 co-localized with hTAC in the patch-like endosomal structures (Fig. S3C), and in the cells containing the tubules, ARF6 co-localized with hTAC in both the patch-like endosomal structures and the tubular endosomal structures (Fig. S4A). We also found that the Rho family GTPase RAC1 co-localized with hTAC in the patch-like endosomal structures (Fig. S3D), and in cells containing the tubules, RAC1 co-localized with hTAC in both the patch-like endosomal structures and the tubular endosomal structures (Fig. S4B). These results imply that both ARF6 and RAC1 participate in hTAC recycling through tubular and patch-like endosomal structures. Further investigation found that the abrogation of ARF6 and RAC1 via constitutively inactive mutants, i.e., ARF6T27N and RAC1T17N, decreased the endocytosis of hTAC (Fig. S3E and S3F), suggesting that ARF6 and RAC1 might be involved in both the endocytosis and recycling of hTAC.

In this study, the APEX2 proximity labeling method was employed for the *in situ* mapping of the potential proteins involved in hTAC endocytosis and trafficking in living cells. Interestingly, a few CDE-related proteins were identified by the hTAC-APEX2 mapping, and the subsequent experiments confirmed the participation of the CDE pathway in hTAC endocytosis. However, we did not exclude the involvement of the CIE pathway in hTAC endocytosis because we demonstrated that hTAC was internalized into EHD1-positive patch-like endosomal structures that were positive for other CIE cargoes but not the CDE cargo. Thus, we speculate that hTAC internalizes via both the CDE and CIE pathways. This complicated endocytosis mechanism involving internationalization via multiple pathways not only occurs in hTAC but also in other PM proteins, including muscarinic acetylcholine receptor 4 (Boucrot et al., 2015; Wan et al., 2015), platelet-derived growth factor receptor β (Jastrzebski et al., 2017) and EGFR (Boucrot et al., 2015; Caldieri et al., 2017). Altogether, these results and our findings expand our understanding of the endocytic characteristics of certain PM protein cargoes.

## NOTES

We would like to thank the Center of Biological Imaging at the Institute of Biophysics, Chinese Academy of Sciences, for assisting with the cell imaging experiments. We are grateful to Yan Teng for assistance with the confocal analysis and to Shuoguo Li for support with the live-cell imaging using SIM. We thank Dr. Junying Jia and Shuang Sun for providing technical assistance with the flow cytometry experiments. We thank Dr. Jifeng Wang for operating the mass spectrometer. We appreciate Dr. Li Zheng for her assistance and discussion concerning the APEX2 technology. This study was supported by grants from Ministry of Science and Technology of the People’s Republic of China (2016YFA0500203), the National Natural Science Foundation of China (Grant Nos. 31770900, 31730054, and 31270884), and the Youth Innovation Promotion Association of the Chinese Academy of Sciences (2011087).

Xinyu Zhu, Min Li, Xiaojun Xu, Rui Zhang, Xiaofei Zhang, Zhuo Ma, Jingze Lu, Tao Xu, Junjie Hou and Eli Song declare that they have no conflict of interest. This article does not contain any studies with human or animal subjects performed by the any of the authors.

## Electronic supplementary material

Below is the link to the electronic supplementary material.

Supplementary material 1 (AVI 2621 kb)

Supplementary material 2 (AVI 480 kb)

Supplementary material 3 (XLSX 214 kb)

Supplementary material 4 (PDF 1140 kb)
