# Supplementary material for: hTAC internalizes via both clathrin-dependent and clathrin-independent endocytosis in mammalian cells
Source: Protein Cell. 2018 Mar 17;9(10):896–901. doi: 10.1007/s13238-018-0508-9 (PMC6160390; doi:10.1007/s13238-018-0508-9)
Supplement: Supplementary file 4 — Supplementary material 4 (PDF 1140 kb) [file 13238_2018_508_MOESM4_ESM.pdf]

Supplemental data includes the materials and methods, four figures, one table and two movies.

MATERIALS AND METHODS

Reagents

Dulbecco's modified Eagle's medium (DMEM), McCoy's 5A (modified) medium, fetal bovine serum (FBS), Lipofectamine 2000, puromycin, the anti-V5 epitope tag antibody, streptavidin magnetic beads (88816), the Alexa Fluor dye-conjugated secondary antibody, Dextran-TMR (D1819), Alexa Fluor 488-Tfn (T13342), Qdot625 secondary antibody conjugates (A10195), the anti-HA tag antibody (26183), puromycin (A1113803), and Alexa Fluor 488-streptavidin were purchased from Thermo Fisher Scientific (Waltham, MA). The anti-CD25 (hTAC) antibody (302602) and anti-CD44 antibody (338802) were purchased from BioLegend (San Diego, CA). The anti-MHC I antibody (ab23755) was purchased from Abcam (Cambridge, MA), and the anti-Myc antibody (PLA0001) was purchased from Sigma (Saint Louis, MO). The A/C heterodimerizer (635057, also referred to as AP21967, which is a rapamycin analogue) was purchased from Clontech (Mountain View, CA). Biotin-Phenol (BP, LS-3500.1000) was purchased from Iris Biotech GmbH (Marktredwitz, DE), and Protein G Sepharose 4 Fast Flow (17061801) was purchased from GE Healthcare (Buckinghamshire, UK).

siRNA, plasmid construction and transfection

The plasmids expressing dynamin2k44a-egfp and CLTA-mCherry were gifts from

Tomas Kirchhausen (Harvard Medical School). The *apex2* gene was purchased from Addgene (Cambridge, MA). The genes for hTAC-apex2 and frbT2098L-HA-hTAC were cloned into the PQCXIP vector. The genes myc-ap180c, bfp-ehd1, v5-rac1, v5-rac1t17n, arf6-v5 and arf6t27n-v5 were individually cloned into the pcDNA3.1 vector. Lipofectamine 2000 was used for the transfection of all plasmids according to the manufacturer's recommendations. Cells transfected with v5-rac1, v5-rac1t17n, arf6-v5 or arf6t27n-v5 were subjected to various assays 12 h after transfection, whereas cells transfected with the remaining genes were subjected to various assays approximately 20 h after transfection.

The siRNA targeting cltc was purchased from Thermo Fisher Scientific; the siRNAs targeting ap2a1 (5'-GAGCAUGUGCACGCUGGCCAGCU-3' (Devadas et al., 2014)) and ap2b1 (5'-GGAUGAGGAUCCCUAUGUUTT-3' were designed and synthesized by GenePharma (Suzhou, CN)). Lipofectamine 2000 was used for the siRNA transfection. Cells transfected with the CLTC-targeted siRNA were subjected to various assays 48 h after transfection; the cells transduced with siRNAs against ap2a1 and ap2b1 required two transfections, with an approximately 24-h interval between the two transfections, and the transfected cells were subjected to various assays 72 h after the first transfection.

Cells and monoclonal cell generation

HeLa cells purchased from Xiangf Bio (Shanghai, China) were cultured in DMEM supplemented with 10% FBS. U2OS cells stored in our laboratory were cultured in McCoy's 5A (modified) medium supplemented with 10% FBS. hTAC-APEX2 was

stably expressed in the U2OS cells via retrovirus infection (Du et al., 2016) with a high infection efficiency, and the uninfected cells were eliminated using 1 μg/ml puromycin. Similarly, FRBT2098L-HA-hTAC stable-expression monoclones were generated in HeLa cells by virus infection and selected using 0.6 μg/ml puromycin. The cells were then sorted by flow cytometry (FACSAria III, BD, Franklin Lakes, NJ) and seeded into a 96-well plate for culturing. The HeLa monoclone with the homogeneous and low expression of FRBT2098L-HA-hTAC (hereafter called hTAC-HeLa) was selected for the subsequent experiments.

Biotin labeling and the enrichment of biotinylated proteins

For the biotin labeling and enrichment of biotinylated proteins, we followed the methods reported by Alice Ting's laboratory (Rhee et al., 2013; Lam et al., 2015; Hung et al., 2016). The samples used for the biotin labeling analysis included two control groups (CGs) and one experimental group (EG). Control group 1 (CG1) comprised wild-type U2OS cells treated with substrates (BP and H_2O_2). The experimental group comprised U2OS cells stably expressing hTAC-APEX2 treated with substrates, and control group 2 (CG2) comprised U2OS cells stably expressing hTAC-APEX2 without the substrate treatment. For the biotin labeling, cells stably expressing hTAC-APEX2 were incubated with 500 μm BP medium for 30 min at 37 ℃; then, 1 mM H_2O_2 (final concentration) was added to the BP medium. The cells were chased for 1 min at room temperature and then immediately quenched by exchanging the BP/H_2O_2 solution with the quencher solution (10 mM sodium azide, 10 mM sodium ascorbate, and 5 mM Trolox in PBS) twice. After the quenching, the

cells were washed with PBS twice to remove the reaction regents. The negative control cells without the addition of BP and H_2O_2 were washed twice with PBS. After washing, the cells were lysed with freshly prepared RIPA lysis buffer (50 mM Tris, 150 mM NaCl, 0.1% SDS, 0.5% sodium deoxycholate, 1% NP40, 1× protease cocktail, 1 mM PMSF, 10 mM sodium azide, 10 mM sodium ascorbate, and 5 mM Trolox) for 30 min on ice. The lysates were clarified by centrifugation at 14,000 rpm for 10 min at 4 ℃. The supernatant was filtered with a 3K filter unit by centrifugation at 12,000 rpm for 10 min at 4 ℃ to remove the free BP. The protein concentration in the supernatant was assayed using a BCA kit. A small portion of each lysate was used for the Western blotting to confirm the biotin labeling reaction, the remaining lysate fraction was used to enrich the biotin-labeled proteins with streptavidin-coated magnetic beads. Before mixing with the lysates, the streptavidin-coated magnetic beads were washed twice with RIPA lysis buffer. For each 2 mg of total protein (protein concentration was approximately 3 mg/mL), 100 μL of streptavidin bead slurry was added. The mixed suspensions were incubated at room temperature for 1 h with gentle rotation. After collecting with a magnetic stand, the streptavidin beads were washed with RIPA lysis buffer twice, 1 M KCl once, 0.1 M Na2CO3 once, 2 M urea in 10 mM Tris-HCl (pH 8.0) once, and again with RIPA lysis buffer twice. Then, the enriched biotinylated proteins were eluted by incubating the beads with 30 μL 1xSDS loading buffer supplemented with 20 mM DTT and heating the mixture to 95 ℃ for 90 s.

In-gel protein digestion and extraction

The enriched biotinylated proteins were separated on a 10% SDS-PAGE gel and stained overnight with Coomassie G-250 (Invitrogen). The lanes of each replicate were manually cut into 3-4 gel bands, and each band was destained with 40% acetonitrile/50 mM NH_4HCO_3. The gels were dehydrated with 100% acetonitrile and dried for 5 min using a SpeedVac. The disulfide bonds were reduced with DTT (10 mM at 56 ℃ for 45 min), and the free sulfhydryl groups were alkylated with iodoacetamide (55 mM) at 25 ℃ for 60 min in the dark. The gel pieces were sequentially washed with 50 mM NH_4HCO_3 and 50% acetonitrile/50 mM NH_4HCO_3 and then dehydrated with 100% acetonitrile. After drying the gels using a SpeedVac, the gels were rehydrated using 100 ng/L trypsin (Promega, V5113) on ice for 30 min, the proteins were digested at 37 ℃ overnight, and the reaction was quenched with 1.0% TFA. The trypsinized peptides were extracted twice with 60% acetonitrile containing 0.1% TFA, and then, the combined digest solution was dried using a SpeedVac.

Mass spectrometry

The NanoLC-MS/MS experiments were performed using a Q Exactive mass spectrometer (Thermo Scientific) coupled to an Easy-nLC 1000 HPLC system (Thermo Scientific). The dried peptides were resuspended in a 0.1% formic acid (FA)/2% acetonitrile solution and loaded onto a 100 m × 2 cm fused silica trap column packed in-house with reversed phase silica (Reprosil-Pur C18 AQ, 5 m, Dr. Maisch, GmbH). The peptides were then separated on an a 75 m × 20 cm C18 column packed with reversed phase silica (Reprosil-Pur C18 AQ, 3 m, Dr. Maisch, GmbH), and the loaded peptides were eluted using a 78-min gradient. Solvent A

comprised 0.1% FA in water, and solvent B comprised 0.1% FA in acetonitrile. The segmented gradient was 4-12% B for 5 min, 12-22% B for 50 min, 22-32% B for 12 min, 32-95% B for 1 min, and 95% B for 7 min at a flow rate of 280 nL/min.

The mass spectrometer was operated in the data-dependent acquisition mode, and full-scan MS data were acquired in the Orbitrap at a resolution of 70,000 (m/z 200) across the mass range of 300-1600 m/z. The target value was 3.00E+06 with a maximum injection time of 60 ms. After the survey scans, the top 20 most intense precursor ions were selected for the MS/MS fragmentation using an isolation width of 2 m/z in the HCD collision cell with an optimized normalized collision energy of 32%. The MS/MS spectra were subsequently acquired in the Orbitrap at a resolution of 17,500 (m/z 200) and a low mass cut-off setting of 100 m/z. The target value was set as 5.00E+04 with a maximum injection time of 80 ms. The dynamic exclusion time was 50 s. The nano-electrospray ion source setting was set as a 2.0 kV spray voltage, no sheath gas flow, and a 320 ℃ heated capillary temperature.

Protein identification and quantification

The raw MS data were processed using Proteome Discovery (version 1.4, Thermo Scientific). Briefly, the peptide identification was performed using the SEQUEST HT search engine against the UniProt database (*Homo sapiens*, 2013.6 (The UniProt, 2017)) supplemented with all frequently observed contaminants in MS. The following parameters were used for the database search: 10 ppm precursor mass tolerance; 0.02 Da fragment ion tolerance; up to two missed cleavages; carbamidomethyl cysteine, BP (361.146 Da) on tyrosine, histone, cysteine and tryptophan; and oxidized

methionine as a variable modification. The peptide confidence was set at a high level (q-value < 0.01) for the peptide filter. The proteins were quantified using the spectral counting method (Singec et al., 2016). A spectral count of zero were treated as 1. Molecules that were considered significantly enriched proteins according to the hTAC-APEX2 labeling were required to meet the following criteria: spectral ratio of experimental vs control should be greater than 2.5, and the relative standard deviation of these ratios should be less than 75% (Table S1). The protein-protein interaction networks were retrieved by submitting the gene symbol to the STRING database (Szklarczyk et al., 2017). A minimum interaction score above 0.4 was required.

The mass spectrometry data were deposited in the ProteomeXchange Consortium via the PRIDE (20) partner repository with the dataset identifier PXD006566 (Account details for referees, username: reviewer09980@ebi.ac.uk, password: 9ApmhYI2).

hTAC endocytosis assays using the LAPREP approach

To examine the co-endocytosis of the antibody-labeled hTAC and FKBP-labeled hTAC, the hTAC-HeLa cells were incubated with the FKBP working solution (1 μM AP21967 with either 2 μM EGFP-FKBP or 5 μM mkate2-FKBP) supplemented with 40 μg/ml antibody against hTAC for 15 min at 37 ℃. Then, the cells were washed with PBS and fixed with 4% PFA. The antibody cargo was immunostained with a fluorescent dye-conjugated secondary antibody. Endocytic EGFP-FKBP/mkate2-FKBP co-localized with the endocytic anti-hTAC antibody exquisitely (Figure S2B-C), and the LAPREP approach was selected for the hTAC

endocytosis assays. Additionally, unlike the antibody applied in the endocytosis assays of PM membrane proteins, in the LAPREP approach, only one FRB molecule can bind to one FKBP molecule, this differs from how the first antibody molecule can bind to various amounts of secondary antibody, which is used to enhance the signal intensity in indirect immunofluorescence experiments. Thus, compared to an antibody-based endocytosis assay, the LAPREP approach is more reasonable for quantifying the endocytic signal from imaging data.

In the hTAC endocytosis assays using the LAPREP approach, the hTAC-HeLa cells were pre-treated with a chemical inhibitor or transfected with a plasmid or siRNA for the indicated time. The FKBP working solution (1 μM AP21967 with either 2 μM EGFP-FKBP or 5 μM mkate2-FKBP) was then added to the cells, and the cells were incubated for 10 min. Then, the cells were chased with cold PBS and washed with a low pH buffer for 1 min to remove some non-endocytic EGFP-FKBP/mKate2-FKBP from the PM. Then, the cells were fixed with 4% PFA, and as needed, certain samples were assessed using immunofluorescence following the protocol described by Wan et al (Wan et al., 2015). Subsequently, images of the samples were acquired under an Olympus FV1200 laser scanning confocal microscope (Tokyo, Japan) with a 100x (NA=1.45) oil objective. Identical microscope settings were used for samples within the same group. The subplasmalemmal plane was selected for further analysis where the signal was most obvious and no longer accessible to externally applied mkate2-FKBP or EGFP-FKBP on the PM. To analyze the percentage of cells containing hTAC tubules, cells that

contained one or more tubules longer than 1.6 μm (equivalent to 10 pixels in our confocal images) were defined as positive.

In assays examining the co-endocytosis of hTAC and CD44/MHC I, an FKBP working solution supplemented with 40 μg/ml antibody against MHC I or 20 μg/ml of the antibody against CD44 was added to hTAC-HeLa cells transfected with BFP-EHD1, and the cells were incubated for 15 min at 37 ℃. The remaining external cargo on the PM was removed with low pH buffer (200 mM NaCl, 50 mM MES, 1 mM $MgCl_2$, and 0.1 mM $CaCl_2$, pH=3.0) for 3 min, and then, the cells were fixed and immunostained with a fluorescent dye-conjugated secondary antibody targeting the primary antibody bound to the cargo protein.

In assays examining the co-endocytosis of hTAC and dextran, hTAC-HeLa cells transfected with BFP-EHD1 were incubated with the FKBP working solution supplemented with 5 mg/ml of dextran-TMR for 10 min at 37 ℃.

The samples used for the co-endocytosis assays were also assessed under an Olympus FV1200 laser scanning confocal microscope (Tokyo, Japan) with a 100x (NA=1.45) oil objective.

Live cell imaging

For the TIRFM imaging, the hTAC-HeLa cells were transfected with clta-mCherry. After approximately 20 h, the cells were incubated with the FKBP working solution (2 μM EGFP-FKBP+1 μM AP21967) for 5 m at 37 ℃. Then, the cells were washed with warm PBS and subjected to imaging in phenol red-free DMEM by TIRFM with a 150x (NA=1.49) oil objective.

In the live cell imaging shown in movie1 and Figure S3A, the focal plane of the targeted BFP-EHD1 expressing cell was selected and fixed by the confocal system. Then, the images were taken before and after the addition of the FKBP working solution (2 μM EGFP-FKBP with 1 μM AP21967) under an FV1200 laser scanning confocal microscope with a 100x (NA=1.45) oil objective.

For the live cell imaging shown in movie2 and Figure S3B, the hTAC-HeLa cells were incubated with the FKBP working solution (2 μM EGFP-FKBP with 1 μM AP21967) for 10 m at 37 ℃ and washed with warm PBS. Then, the cells were subjected to image capture in DMEM without phenol red using a DeltaVision OMX V3 system (GE Healthcare, Little Chalfont, UK) in wide-field mode with a 100x (NA=1.40) oil objective. The images were deconvoluted, aligned and reconstructed using the softWoRx software (GE Healthcare).

Protein purification, immunoprecipitation and Western blotting

For the protein purification, the methods used for the generation of the constructs pRSETa-EGFP-FKBP and pRSETa-mkate2-FKBP and protein purification were described previously (Zhang et al., 2014).

For the co-immunoprecipitation, the HeLa-hTAC cells were lysed using lysis buffer (50 mM Tris-HCl, pH 8.0, 150 mM NaCl, 5 mM EDTA, 0.05% NP-40, 0.1% SDS, and protease inhibitors) on ice for 30 min. The lysates were pre-cleared by centrifugation at 140,000 rpm for 10 min at 4 ℃. The protein concentration in the supernatant was assayed using a BCA kit. A total protein content of 7.5 mg from each sample was mixed with 5 μg of the anti-HA tag antibody. The mixture was incubated

for 6 h at 4 ℃ with gentle rotation. Before being added to the mixture, the Protein G beads were blocked with 5% BSA with gentle rotation for 3 h at 4 ℃. Then, the supernatant-antibody-Protein G solution was incubated for 2 h at 4 ℃ with gentle rotation, and the Protein G beads were washed with washing buffer (50 mM Hepes, pH 7.44, 150 mM NaCl, 1 mM EDTA, and protease inhibitors) four times. The bound proteins were eluted with SDS-PAGE buffer by incubating the sample for 3 min at 95 ℃. The immunoprecipitates were subjected to Western blotting as previously described (Wang et al., 2011) using the indicated antibody.

EM sample preparation and imaging

hTAC-HeLa cells grown on ACLAR film were incubated with 40 μg/mL anti-hTAC antibody for 15 min at 37 ℃, fixed with 4% PFA and immunostained with a Qdot625-conjugated secondary antibody. Then, the cells were rinsed with PBS and post-fixed with 1% osmium tetroxide for 1 h at room temperature. After rinsing with distilled water, the cells were stained en bloc with 1% aqueous uranyl acetate overnight at 4 ℃. After dehydrating using an ethanol-graded series, the cells were embedded and polymerized in EMbed-812 resin at 60 ℃ for 48 h. Ultrathin sections (70 nm) were cut using an ultramicrotome (Leica EM UC6, Wetzlar, Germany) and placed on Formvar-coated grids. The TEM images were observed and recorded using an FEI Spirit (Hillsboro, OR) at 120 kV.

Figure S1: APEX2-based proteomic mapping of proteins in the vicinity of hTAC in living cells.

(A) A schematic diagram of hTAC-APEX2 depicting the genetically engineered peroxidase APEX2 tagged to the C-terminus of hTAC. Labeled proteins are within approximately 20 nm of the tag. Certain proteins are present in complexes with hTAC in living cells.

(B) The fusion of APEX2 to hTAC did not affect hTAC endocytosis. Scale bar, 10 μm.

(C) Biotin-tagged proteins induced by hTAC-APEX2-based biotin-phenol labeling were detected under the PM or in the cytosol as expected. In addition, the immunofluorescence experiment showed that hTAC-APEX2 correctly localized on the PM or in the cytosol. Scale bar, 10 μm.

(D) Immunoblotting of biotinylated proteins by streptavidin conjugated to horseradish peroxidase (streptavidin-HRP). SDS-PAGE gel showing the cell lysates of U2OS cells stably expressing hTAC-APEX2 incubated in the presence (EG) or absence of substrates (CG2) and wild-type U2OS cells treated with substrates (CG1). CG1/2 indicates control groups 1 and 2, respectively; EG indicates the experimental group.

(E) Immunoblotting of streptavidin bead-enriched samples shown in (D). Two independent replicates were analyzed.

Figure S2: Detection of hTAC endocytosis using the LAPREP approach or an antibody-labelling-based method.

(A) A schematic diagram of the LAPREP approach for the detection of hTAC endocytosis.

(B-C) Antibody-labeled hTAC and fluorescent protein-tagged FKBP labeled hTAC co-localized exquisitely in the cytosol, and no signal of fluorescent protein-tagged FKBP was detected in the cells incubated with the fluorescent protein-tagged FKBP in the absence of AP21967, demonstrating that the LAPREP approach was able to specifically detect hTAC endocytosis similarly to the method based on antibody labelling. Scale bar, 10 μm.

Figure S3: Dynamic nature of the patch-like endosomal structures and potential roles of the GTPases ARF6 and RAC1 in hTAC endocytosis and trafficking.

(A) Live cell imaging showing the rapid internalization of hTAC into patch-like endosomal structures (arrowheads). The red arrowhead indicates the time point at which the EGFP-FKBP working solution was added. Scale bar, 10 μm.

(B) Dynamic images of rich tubulo-vesicular structures continuously budding from and fusing to other structures. Scale bar: 10 μm in Figure B', 1 μm in Figure B", and 0.5 μm in Figure B"'.

(C-D) Endocytic hTAC co-localized with ARF6-V5/V5-RAC1 in the BFP-EHD1-positive patch-like structures (arrowheads). Scale bar, 10 μm.

(E-F) hTAC endocytosis was decreased in the ARF6T27N-V5/V5-RAC1T17N-transfected cells. The left column shows a representative image of the experiment, and the right column shows the quantification

of the assay. Three independent replicates were performed. For ARF6-V5, N=113/84 (control/ARF6-V5) cells and for V5-RAC1, N=101/97 (control/V5-RAC1) cells. *** indicates $p<0.001$, and ** indicates $p<0.01$, Student's *t*-test. Scale bar, 10 μm.

Figure S4: Co-localization of hTAC and ARF6-V5/V5-RAC1 in cells containing tubules.

(A-B) Endocytic hTAC co-localized with ARF6-V5/V5-RAC1 in the BFP-EHD1-positive patch-like endosomal structures and BFP-EHD1-positive tubules (arrowheads) in the cells containing tubules. Scale bars, 10 μm.

Table S1. Proteins identified by the hTAC–APEX2 mapping.

Movie 1: hTAC rapidly internalizes into the EHD1-positive patch-like endosomal structures.

Movie showing the detection of endocytic hTAC in the EHD1-positive patch-like endosomal structures in less than 15 s after adding the FKBP working solution (t=0 s). Time interval between frames is 2.5 s. Playback is 2 frames per second (fps). Scale bar, 10 μm. See also Figure S3A.

Movie 2: Patch-like endosomal structures are highly dynamic.

Movie showing the rich tubulo-vesicular structures (arrowheads) quickly and continuously budding from and fusing to other structures. Time interval between

frames is 0.4 s. Playback is 1 frame per second (fps). Scale bar, 1 μm. See also Figure S3B.

Figure S1:

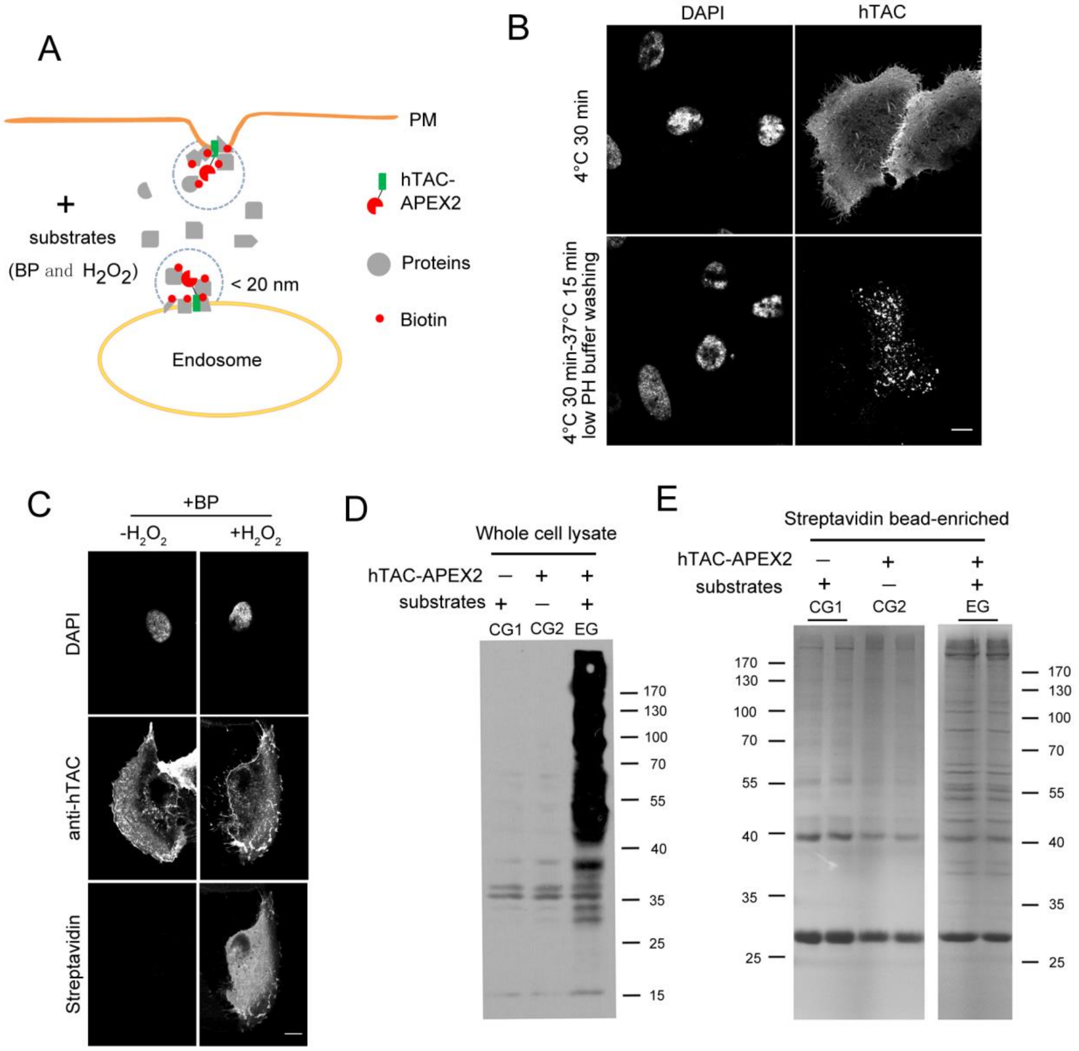

A
PM
+
substrates
(BP and H2O2)
< 20 nm
Endosome
hTAC-APEX2
Proteins
Biotin
B
DAPI
hTAC
4°C 30 min
4°C 30 min-37°C 15 min low PH buffer washing
C
+BP
-H2O2
+H2O2
DAPI
anti-hTAC
Streptavidin
D
Whole cell lysate
hTAC-APEX2
substrates
CG1 CG2 EG
170
130
100
70
55
40
35
25
15
E
Streptavidin bead-enriched
hTAC-APEX2
substrates
CG1 CG2 EG
170
130
100
70
55
40
35
25

Figure S2:

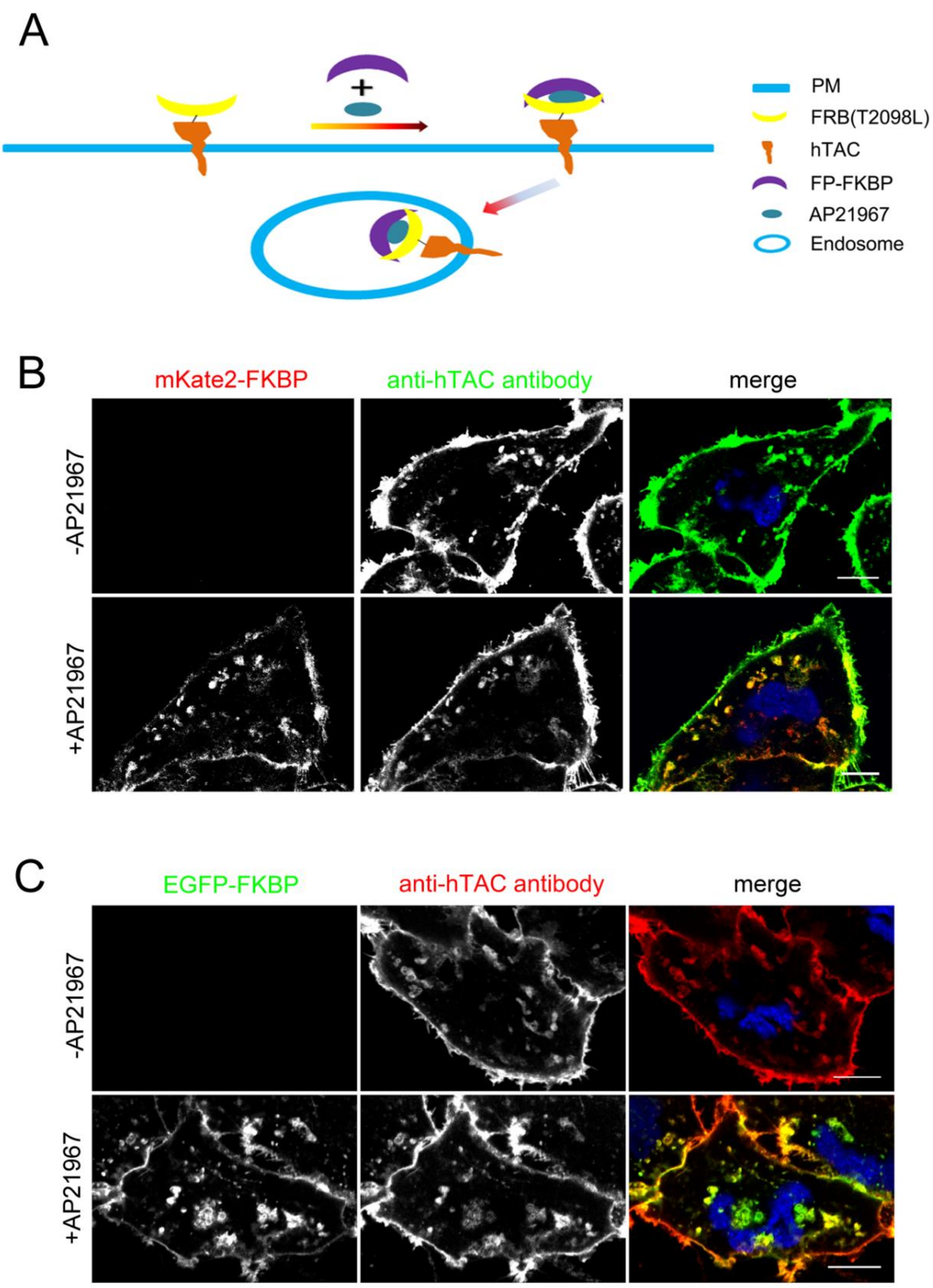
A
+
PM
FRB(T2098L)
hTAC
FP-FKBP
AP21967
Endosome
B
mKate2-FKBP
anti-hTAC antibody
merge
-AP21967
+AP21967
C
EGFP-FKBP
anti-hTAC antibody
merge
-AP21967
+AP21967

Figure S3:

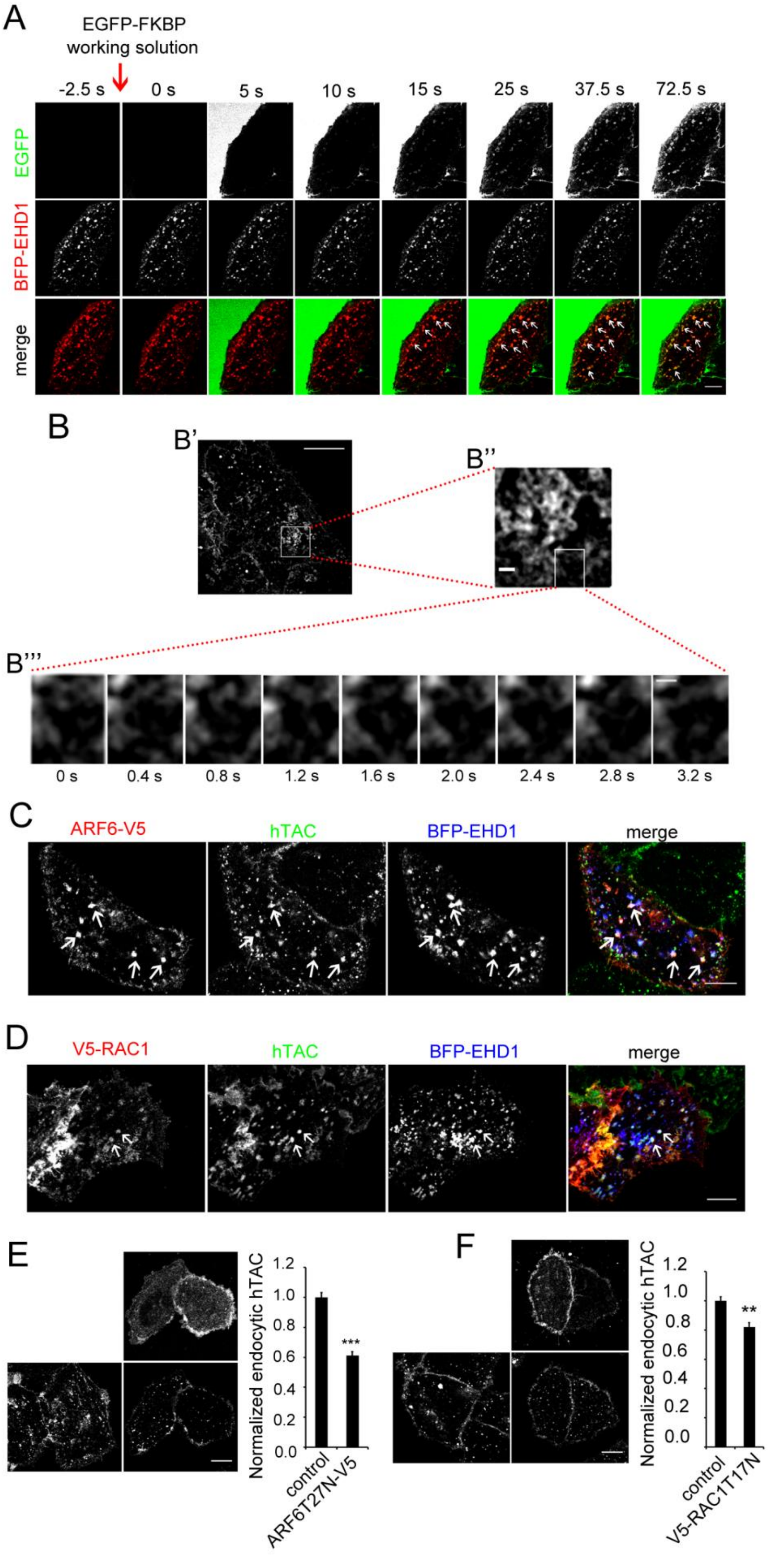
A
EGFP-FKBP
working solution
-2.5 s
0 s
5 s
10 s
15 s
25 s
37.5 s
72.5 s
EGFP
BFP-EHD1
merge
B
B'
B''
B'''
0 s
0.4 s
0.8 s
1.2 s
1.6 s
2.0 s
2.4 s
2.8 s
3.2 s
C
ARF6-V5
hTAC
BFP-EHD1
merge
D
V5-RAC1
hTAC
BFP-EHD1
merge
E
Normalized endocytic hTAC
1.2
1.0
0.8
0.6
0.4
0.2
0.0
***
control
ARF6T27N-V5
F
Normalized endocytic hTAC
1.2
1.0
0.8
0.6
0.4
0.2
0.0
**
control
V5-RAC1T17N

Figure S4:

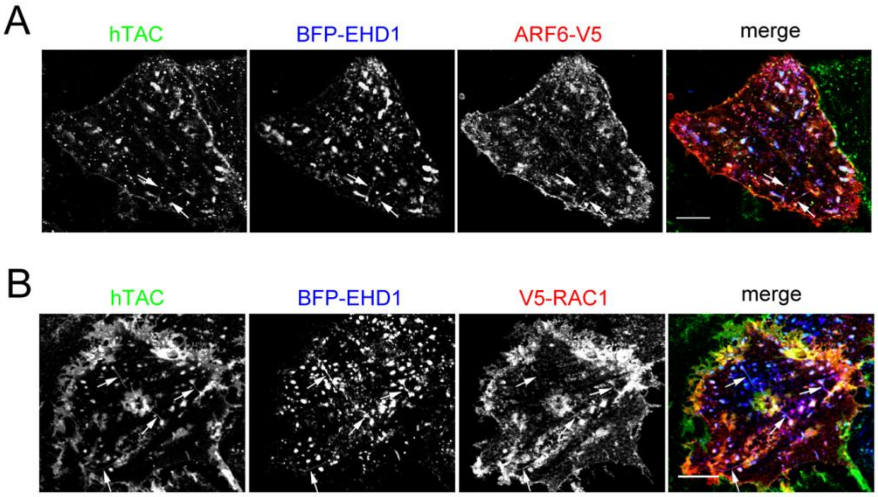

Movie1:

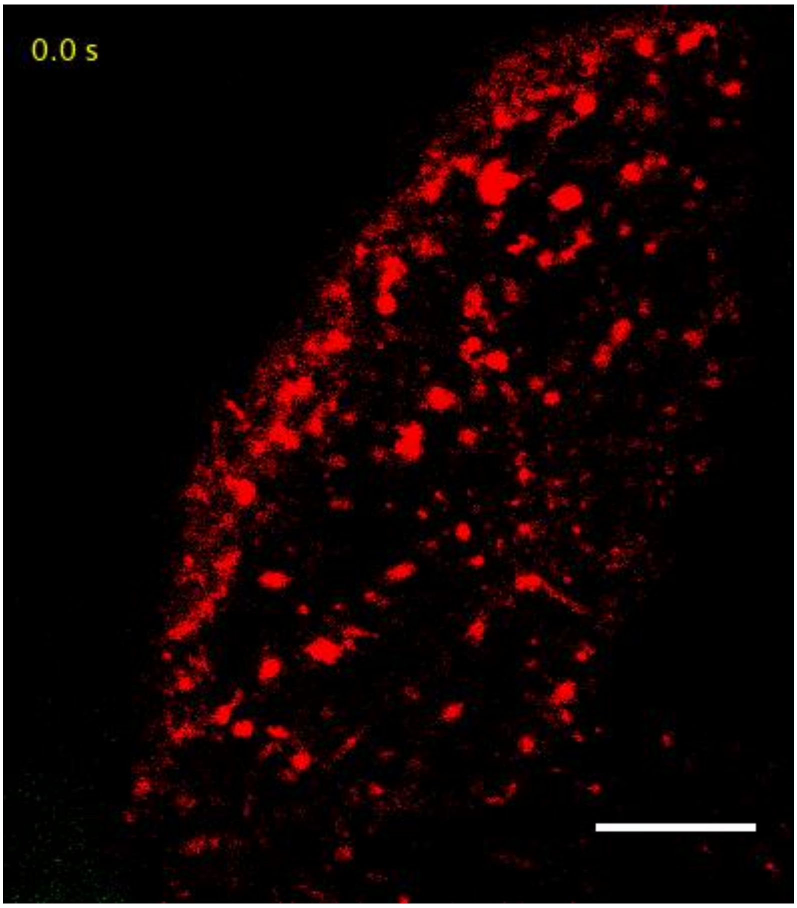

Movie2:

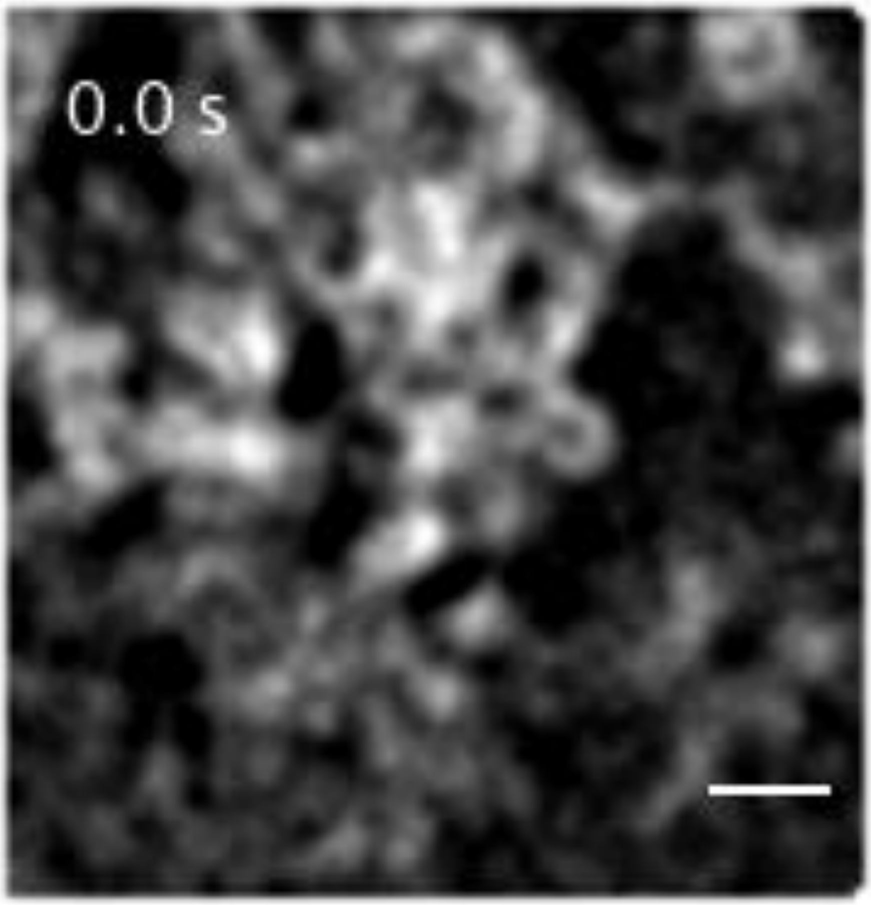

Reference：

Devadas, D., Koithan, T., Diestel, R., Prank, U., Sodeik, B., and Dohner, K. (2014). Herpes simplex virus internalization into epithelial cells requires Na+/H+ exchangers and p21-activated kinases but neither clathrin- nor caveolin-mediated endocytosis. J Virol 88, 13378-13395.

Du, W., Zhou, M.G., Zhao, W., Cheng, D.W., Wang, L.F., Lu, J.Z., Song, E.L., Feng, W., Xue, Y.H., Xu, P.Y., *et al.* (2016). HID-1 is required for homotypic fusion of immature secretory granules during maturation. Elife 5.

Hung, V., Udeshi, N.D., Lam, S.S., Loh, K.H., Cox, K.J., Pedram, K., Carr, S.A., and Ting, A.Y. (2016). Spatially resolved proteomic mapping in living cells with the engineered peroxidase APEX2. Nat Protoc 11, 456-475.

Lam, S.S., Martell, J.D., Kamer, K.J., Deerinck, T.J., Ellisman, M.H., Mootha, V.K., and Ting, A.Y. (2015). Directed evolution of APEX2 for electron microscopy and proximity labeling. Nat Methods 12, 51-54.

Rhee, H.W., Zou, P., Udeshi, N.D., Martell, J.D., Mootha, V.K., Carr, S.A., and Ting, A.Y. (2013). Proteomic mapping of mitochondria in living cells via spatially restricted enzymatic tagging. Science 339, 1328-1331.

Singec, I., Crain, A.M., Hou, J., Tobe, B.T., Talantova, M., Winquist, A.A., Doctor, K.S., Choy, J., Huang, X., La Monaca, E., *et al.* (2016). Quantitative Analysis of Human Pluripotency and Neural Specification by In-Depth (Phospho)Proteomic Profiling. Stem Cell Reports 7, 527-542.

Szklarczyk, D., Morris, J.H., Cook, H., Kuhn, M., Wyder, S., Simonovic, M., Santos, A., Doncheva, N.T., Roth, A., Bork, P., *et al.* (2017). The STRING database in 2017: quality-controlled protein-protein association networks, made broadly accessible. Nucleic Acids Res 45, D362-D368.

The UniProt, C. (2017). UniProt: the universal protein knowledgebase. Nucleic Acids Res 45, D158-D169.

Wan, M., Zhang, W., Tian, Y., Xu, C., Xu, T., Liu, J., and Zhang, R. (2015). Unraveling a molecular determinant for clathrin-independent internalization of the M2 muscarinic acetylcholine receptor. Sci Rep 5, 11408.

Wang, L.F., Zhan, Y., Song, E.L., Yu, Y., Jiu, Y.M., Du, W., Lu, J.Z., Liu, P.S., Xu, P.Y., and Xu, T. (2011). HID-1 is a peripheral membrane protein primarily associated with the medial- and trans-Golgi apparatus. Protein & Cell 2, 74-85.

Zhang, X., Deng, Y., Chang, H., Ji, C., Zhang, M., Peng, J., Xu, T., and Xu, P. (2014). The specific and rapid labeling of cell surface proteins with recombinant FKBP-fused fluorescent proteins. Protein Cell 5, 800-803.
